# Descriptive analysis of national bovine viral diarrhoea test data in England (2016–2020)

**DOI:** 10.1002/vetr.1854

**Published:** 2022-07-25

**Authors:** Naomi S. Prosser, Edward M. Hill, Derek Armstrong, Lorna Gow, Michael J. Tildesley, Matt J. Keeling, Jasmeet Kaler, Eamonn Ferguson, Martin J. Green

**Affiliations:** ^1^ School of Veterinary Medicine and Science University of Nottingham Sutton Bonington UK; ^2^ Zeeman Institute for Systems Biology & Infectious Disease Epidemiology Research School of Life Sciences and Mathematics Institute University of Warwick Coventry UK; ^3^ Joint Universities Pandemic and Epidemiological Research UK; ^4^ BVDFree England Scheme Stoneleigh Park Kenilworth UK; ^5^ Agriculture and Horticulture Development Board Stoneleigh Park Kenilworth UK; ^6^ School of Psychology University of Nottingham Nottingham UK

**Keywords:** antibody testing, bovine viral diarrhoea, BVDFree England, disease eradication scheme, virus testing

## Abstract

**Background:**

Bovine viral diarrhoea virus (BVDV) causes substantial economic losses to the cattle industry; however, control and eradication can be achieved by identifying and removing persistently infected cattle from the herd. Each UK nation has separate control programmes. The English scheme, BVDFree, started in 2016 and is voluntary.

**Methods:**

We analysed the test results submitted to BVDFree from 5847 herds between 2016 and 2020.

**Results:**

In 2020, 13.5% of beef breeders and 20.0% of dairy herds that submitted tests had at least one positive (virus/antibody) test result. Although lower than in previous years, there was no clear trend in the proportion of positive tests over time. In virus testing herds, 0.4% of individual tests were positive in 2020, and 1.5% of individual tests were positive in BVDV‐positive virus testing herds. Dairy herds and larger herds were more likely to join BVDFree, and dairy herds were also more likely to virus test than beef breeder herds. Larger herds, herds that used virus testing and herds that had BVDV‐positive test results were more likely to continue submitting tests to BVDFree.

**Conclusions:**

The findings provide a benchmark for the status of BVDV control in England; continued analysis of test results will be important to assess progress towards eradication.

## INTRODUCTION

Bovine viral diarrhoea virus (BVDV) is a pestivirus that affects cattle and is endemic in the UK. BVDV infections in a herd cause diverse production losses, such as reduced fertility, decreased milk yield, abortions, increased susceptibility to other diseases, poor growth rates and mucosal disease.^1^, ^2^ Infection is largely maintained by the presence of persistently infected (PI) animals, which are created when a pregnant cow becomes infected prior to immunocompetence in the developing calf (within the first 120–125 days of gestation).^3^ PI animals shed BVDV their entire life, whereas immunocompetent cattle infected with BVDV are only transiently infected and develop long‐lasting immunity.^4^ In 2021, the Ruminant Health and Welfare Group suggested a target to eradicate BVDV from the UK by 2031.^5^

In order to control and eradicate BVDV, PI animals must be identified and removed from the herd.^6^ There are two strategies commonly used for BVDV detection in herds: virus or antibody testing.^7^, ^8^ Individual viral or antigen tests are used to detect BVDV‐infected animals and are usually conducted as tissue tests, often incorporated into the identification tag. For antibody testing, a sample of unvaccinated cattle from specific management groups (commonly each individual group at 9–18 months of age) are tested for exposure to BVDV (commonly called a check test). Individual antigen or virus testing of the whole group or herd is required following a positive antibody test to identify any animals PI with BVDV. Antigen and virus tests are unable to distinguish between transient and persistent infections; therefore, follow‐up testing is recommended to confirm a PI animal.^9^

A number of countries have had success in BVDV control and eradication using both virus and antibody testing regimens.^10^ BVDV control in the UK is conducted by schemes in each of the home nations, which are diverse in terms of testing used, mandatory participation and progress.^11^ A voluntary scheme commenced in Scotland in 2010 before becoming mandatory in 2013.^12^ In this scheme, farmers could use either an antibody screen or virus test all calves.^12^ The prevalence of BVD in Scotland declined between 2010 and 2019, from 40% to 10% of herds.^13^ Northern Ireland's scheme, which uses virus testing (via ear tag) on every calf, was voluntary when introduced in 2013 and became mandatory in 2016. There was a decrease in herd‐level prevalence from 11% to 4.17% from 2016 to 2021.^14^, ^15^ Wales has a voluntary scheme (Gwaredu BVD), funded by the Welsh Government's Rural Development Programme, that started in 2017 and offers free youngstock antibody screening, with limited additional funding for identifying PI animals.^16^ Changes in BVD prevalence over the first 3 years (to 2020) have not been published for Gwaredu BVD. Elsewhere in the British Isles, Ireland has reduced its herd‐level prevalence of BVD following the introduction of a compulsory programme (tissue tagging of all calves born since 1 January 2013), with an estimated prevalence of BVD in herds of 11.3% at the start of 2013, reducing to 0.55% in 2020.^17^ BVD control became compulsory in the Isle of Man in 2014, and no PI cattle have been detected since 2018.^18^

BVDFree England (hereafter referred to as BVDFree) is the voluntary BVD eradication programme for England that started in 2016.^19^ Members of BVDFree are required to upload the results of all testing for BVD to the BVDFree database. Two years of testing is required in order to achieve a negative status, which can be achieved through either an antigen/virus test (via ear tag) of every newborn calf or blood antibody tests of a minimum of five cattle between 9 and 18 months old from each management group.^20^ In antibody testing herds, follow‐up tests with tissue or blood tests for antigen or virus are needed to identify any PIs in antibody‐positive herds.^20^ Herds may also submit bulk milk test results (antigen, virus or antibody) to BVDFree in addition to the required tests. A parallel scheme (Stamp It Out) ran from 2018 to 2020 and funded farmers to test for BVD, largely using the BVDFree protocols, with the option to also submit the results to BVDFree for recognised BVD‐free herd status.^11^

Assessment of test results and demographics of farmers participating in BVDFree are needed to assess the scheme's progress and inform decisions on further development towards eradication. In this study, we present a descriptive analysis of participation and test results submitted to the BVDFree database between 2016 and 2020. We also investigate whether there is evidence for specific factors impacting the likelihood of herds not continuing to submit tests to the BVDFree scheme, which may offer insights to inform strategies to enhance engagement.

## MATERIALS AND METHODS

### Data source and cleaning

We conducted all data preparation and analysis using R statistical software (version 4.0.5).^21^

BVDFree provided data that included all test results (535,332) from samples submitted between 2016 and 2020 (correct as of 14 April 2021). All herd‐level information associated with each test was collected at the time the herd signed up to BVDFree and included the County Parish Holding number, postcode, type of herd (any combination of dairy/beef breeder/beef finisher/calf rearer), herd size (number of breeding cows) and veterinary practice. We removed the variable ‘herd size’ for 49 (of 5611) herds that gave either implausible herd sizes or a range of sizes. Test‐level information was complete for all tests and included the dates the sample was taken, tested and uploaded, the type of sample (tissue/blood/milk), whether the sample was pooled from multiple animals, the type of test (reported as antibody ELISA, antigen ELISA, antigen PCR and virus PCR), and the laboratory and the test result. We omitted 89 (of 535,332) tests that had a later sample date than the automatically generated test result upload date, resulting in a dataset with 535,243 tests from 5611 herds. Herd‐level details contained missing data: five (<0.01%) tests had a missing County Parish Holding number, 49,402 (9.2%) tests had a missing postcode, 58,678 (11.0%) did not give the type of cattle on the farm, 64,284 (12.0%) tests did not give a herd size and 49,529 (9.3%) tests did not state a veterinary practice.

### Descriptive analysis

To enable the identification of tests in the database that were likely to be from herds conforming to BVDFree testing requirements,^20^ we categorised herds according to the test regimen employed in any one calendar year: virus (only used individual antigen or virus tests) or antibody (minimum of five individual antibody tests, with or without additional virus tests). We then classified herds as virus positive if at least one individual antigen or virus test was positive and antibody positive if at least one individual antibody test was positive. We investigated the testing behaviour of herds that we categorised as using either testing regimen to verify that their testing behaviour appeared compliant with the required test regimen. We also investigated the proportion of herds with a positive virus test for different numbers of individual virus tests as a proportion of their herd size to assess the impact of using different cutoffs in our definition.

Summary statistics were calculated by test regimen (virus or antibody), herd type (beef breeder only or dairy only) and calendar year. Assessment of the spatial distribution of the herds in the dataset by year was conducted by plotting herd locations by postcode. Regional variation in the prevalence of herds with positive virus or antibody test results was assessed by calculating the proportion of the virus or antibody testing herds with a positive test result in each region of England and plotting heatmaps of the resulting prevalence.

### Test regimens used by the participating farmers

Factors associated with the test regimen used by each herd (antibody or virus) were explored using a generalised logistic mixed‐effects model to account for herds testing for multiple years. The model was built using the lme4 package (v1.1‐26).^22^ Herd size had a log‐normal distribution, so we applied a natural log transformation. The fixed effects tested were year of testing, herd size (natural log transformed) and herd type (beef breeder/dairy). Herd was a random effect. Terms with a *p*‐value less than 0.05 were selected in the final model. We assessed the final model fit using decile plots of the observed and predicted data.^23^

### Continued participation of herds in the BVDFree scheme

Factors associated with the herds in the scheme each year participating again the following year were investigated using a generalised logistic mixed‐effects model to account for herds testing for multiple years. The model was built using the lme4 package (v1.1‐26)^22^ and with a natural log transformation to herd size. The fixed effects were year of testing, herd size (natural log transformed), test regimen (virus/antibody) and herd type (beef breeder/dairy). Herd was a random effect. All terms with a *p*‐value < 0.05 were omitted from the final model. Assessment of the final model fit was carried out using decile plots of the observed and predicted data.^23^

## RESULTS

### Participating herd summary statistics

**FIGURE 1:**
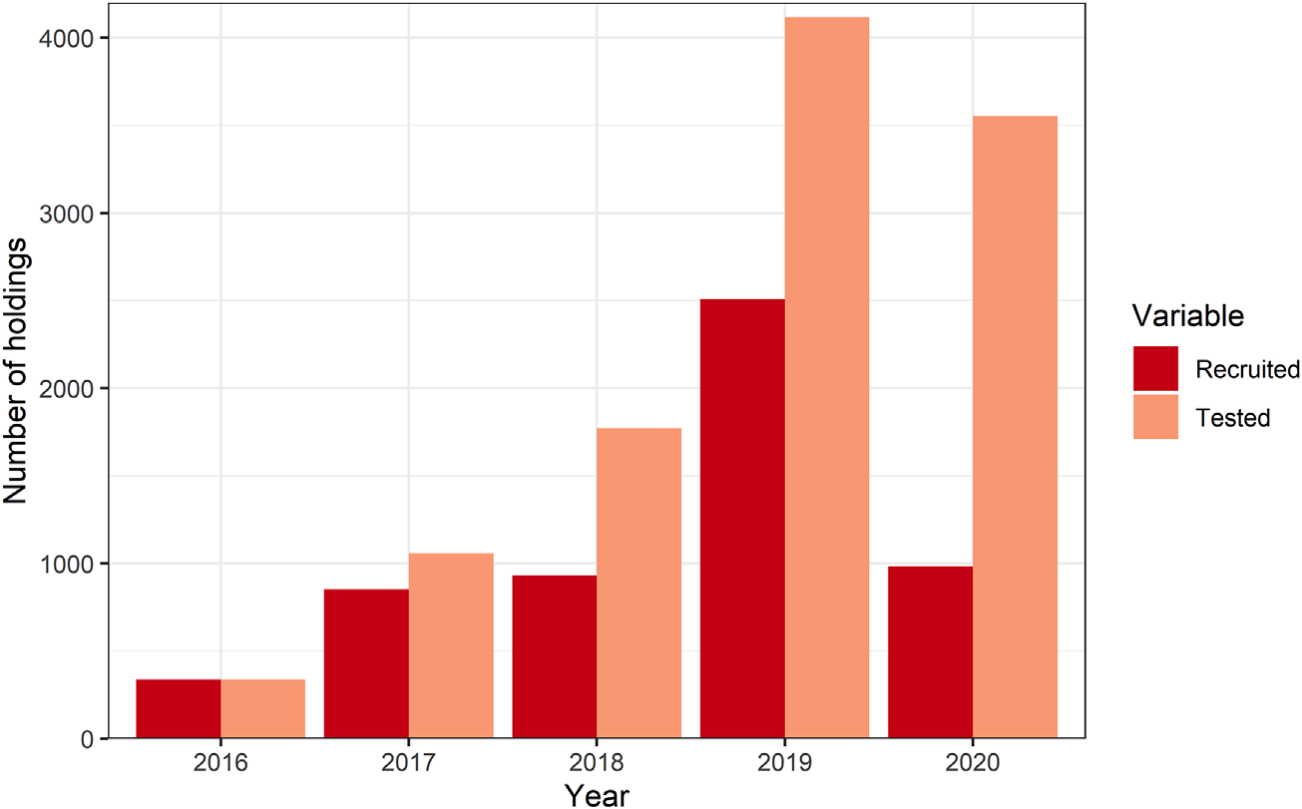
Number of holdings recruited to BVDFree for the first time (recruited) and total number of holdings submitting test results to BVDFree (tested) per calendar year. There are 5611 holdings in total

A total of 5611 herds submitted 535,243 tests to BVDFree between 2016 and 2020 (Figure 1), which represents approximately 15% of English herds.^24^ The number of herds submitting tests increased each year from 2016, peaking at 4118 in 2019 (Figure 1). In 2020, the number of herds submitting tests fell to 3554, corresponding to 63.3% of herds registered with BVDFree. The herds were associated with 264 veterinary practices in total, and the number of veterinary practices participating increased year on year, with 252 submitting tests in 2020.

**FIGURE 2:**
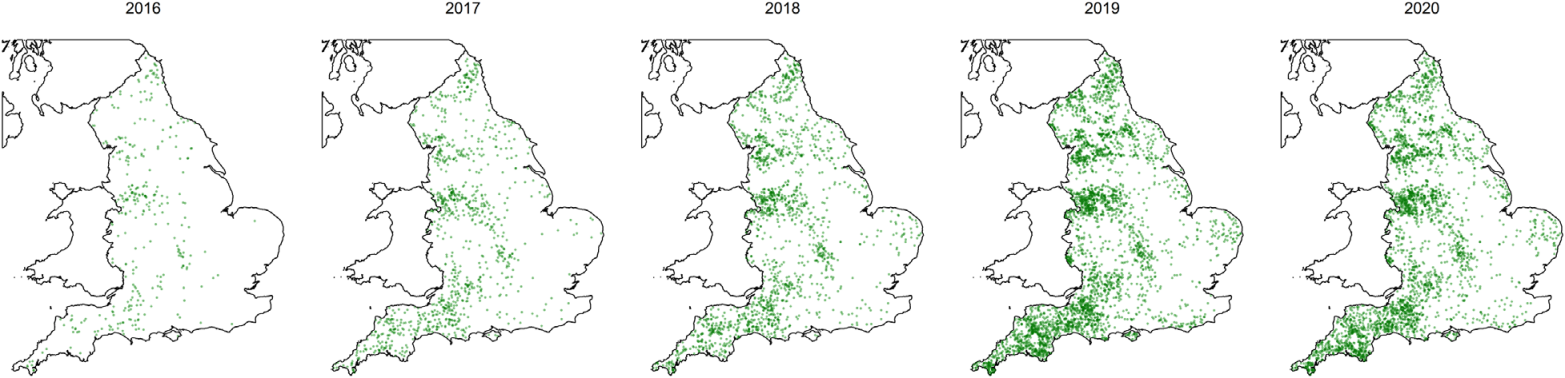
Locations of 5611 herds submitting tests to BVDFree each year for 2016–2020

The median herd size between 2016 and 2020 was 180 (interquartile range [IQR] = 120–280) and 45 (IQR = 24–76) breeding cows for dairy and beef breeding herds, respectively, which is higher than the national averages.^24^ There was little change in herd sizes submitting tests each year, with medians of 170–190 for dairy and 43–50 for beef breeders for 2016–2020. Of the 4386 herds of known herd type, 52.2% (2288) had beef cows, 48.7% (2138) had dairy cows, 22.8% (999) finished beef and 3.3% (144) reared calves (herds could have more than one type of cattle present). These numbers represent a larger proportion of dairy holdings (approximately 20%) than beef holdings (approximately 10%).^24^ In total, 76.2% (3340) of herds were only one type of herd (beef breeder/dairy/beef finisher/calf rearer), of which 51.5% (1720) were dairy breeders and 43.5% (1452) were beef breeders.

Visual inspection of herd locations plotted by postcode in each calendar year showed a similar distribution to the distribution of herds nationally across all years (Figure 2), which was similar to the national distribution of bovine holdings.^25^

The number of new herds recruited to the scheme each year increased each year until 2019 (2507 herds) (Figure 1). There were changes in the types of herds recruited each year, with the proportion of recruited herds that were dairy decreasing each year from 0.68 in 2016 to 0.46 in 2020. There was no trend in beef herd sizes by year; however, there was a decrease in the median size of newly recruited dairy herds each year (except for 2016, with a median of 170 cows), falling from 200 cows in 2017 to 160 cows in 2020.

### Testing regimens used by participating farmers

There were 2368 herds that carried out virus testing for at least one calendar year (4081 herd‐years for 2016–2020). There was a bimodal distribution to the number of tests carried out as a proportion of the number of breeding cows (used as a proxy for the proportion of calves tested), with peaks at both 0.1 and 0.8 (Figure S1). The highest peak was at 0.8 for beef breeders and 0.1 for dairy breeders. Herds with a greater proportion of virus tests were more likely to have a BVD‐positive test result; however, there was only a large change in the proportion of herds positive for BVD when the cutoff for the number of tests as a proportion of herd size was above 0.8 for dairy herds (Figure S2). It was notable that dairy herds were less likely to have a high number of tests as a proportion of herd size (only 26.4% of dairy herd/year combinations had a proportion of tests of at least 0.8 of the herd's size), so we included all herds in estimates of prevalence (see below).

There were 3162 herds that were tested using blood antibody tests for at least 1 year (4519 herd‐years for 2016–2020). Antibody testing herds had a median of 1 test visit/year (range = 1–4 for beef breeders and 1–3 for dairy herds). Thirty percent of antibody testing herds with a positive result also carried out individual virus tests in the same year, and of these, 12.4% had a positive test result with the individual virus testing.

The generalised logistic mixed‐effects model of variables associated with virus versus antibody testing indicated that herds were more likely to antibody test in 2018–2020 than in 2016 (Table 1). Dairy herds were more likely to virus test than beef breeder herds (odds ratio = 7.18, 95% confidence interval = 4.49–11.50). Herd size was not selected in the final model. Model fit was deemed adequate (Figure S3).

**TABLE 1:** Results of a generalised logistic mixed‐effects model to identify factors associated with virus testing (compared with antibody testing) for bovine viral diarrhoea virus in 2746 cattle herds from 2016 to 2020 (a total of 5139 herd‐years)

	All herds		Virus testing herds				
Fixed effects	*N*	%	*N*	%	OR	95% CI	*p*‐Value
Year of test							
2016	189	3.7	137	72.5	1.00		
2017	513	10.0	349	68.0	0.73	0.35–1.51	0.390
2018	891	17.3	464	52.1	**0.24**	**0.12–0.48**	**<0.001**
2019	1762	34.3	493	28.0	**0.04**	**0.02–0.08**	**<0.001**
2020	1784	34.7	886	49.7	**0.30**	**0.15–0.58**	**<0.001**
Herd type							
Beef	2591	50.4	960	37.1	1.00		
Dairy	2548	49.6	1369	53.7	**7.18**	**4.49–11.50**	**<0.001**

*Note*: *N* = number of herd/year combinations, % = percent of herd/year combinations that are in a fixed effect from all herd/year combinations (all herds) and that are virus tested from all herd/year combinations in a fixed effect (virus testing herds). Significant terms (*p* < 0.05) are in bold.

Abbreviations: OR, odds ratio; 95% CI, Wald's 95% confidence interval.

**TABLE 2:** Results of a generalised logistic mixed‐effects model to identify factors associated with submitting (as opposed to not submitting) bovine viral diarrhoea tests to BVDFree the following year from 2016 to 2019 in 2222 herds (a total of 3355 herd‐years)

	All herds		Herds testing the next year				
Fixed effects	*N*	%	*N*	%	OR	95% CI	*p*‐Value
Number of years submitting tests	3359	100.0	3359	100.0	**1.95**	**1.72–2.23**	**<0.001**
Proportion of years positive	3359	100.0	3359	100.0	**1.33**	**1.08–1.65**	**0.009**
ln(herd size)	3359	100.0	3359	100.0	**1.24**	**1.15–1.34**	**<0.001**
Year							
2016	189	5.6	122	64.6	1.00		
2017	513	15.3	409	79.7	**2.11**	**1.44–3.09**	**<0.001**
2018	892	26.6	712	79.8	**2.09**	**1.45–3.01**	**<0.001**
2019	1765	52.5	1137	64.4	1.10	0.78–1.55	0.584
Type of testing							
Antibody	1915	57.0	1179	61.6	1.00		
Virus	1444	43.0	1201	83.2	**2.44**	**2.02–2.97**	**<0.001**

*Note*: *N* = number of herd/year combinations, % = percent of herd/year combinations that are in a fixed effect from all herd/year combinations (all herds) and that test the next year from all herd/year combinations in a fixed effect (herds testing the next year). OR (for continuous fixed effects this is for an additional year submitting tests, for a change in proportion of years positive from 0 to 1, and for an increase in 1 of the natural log of the herd size). Significant terms (*p* < 0.05) are in bold.

Abbreviations: OR, odds ratio; 95% CI, Wald's 95% confidence interval.

### Prevalence of virus‐ or antibody‐positive herds

**FIGURE 3:**
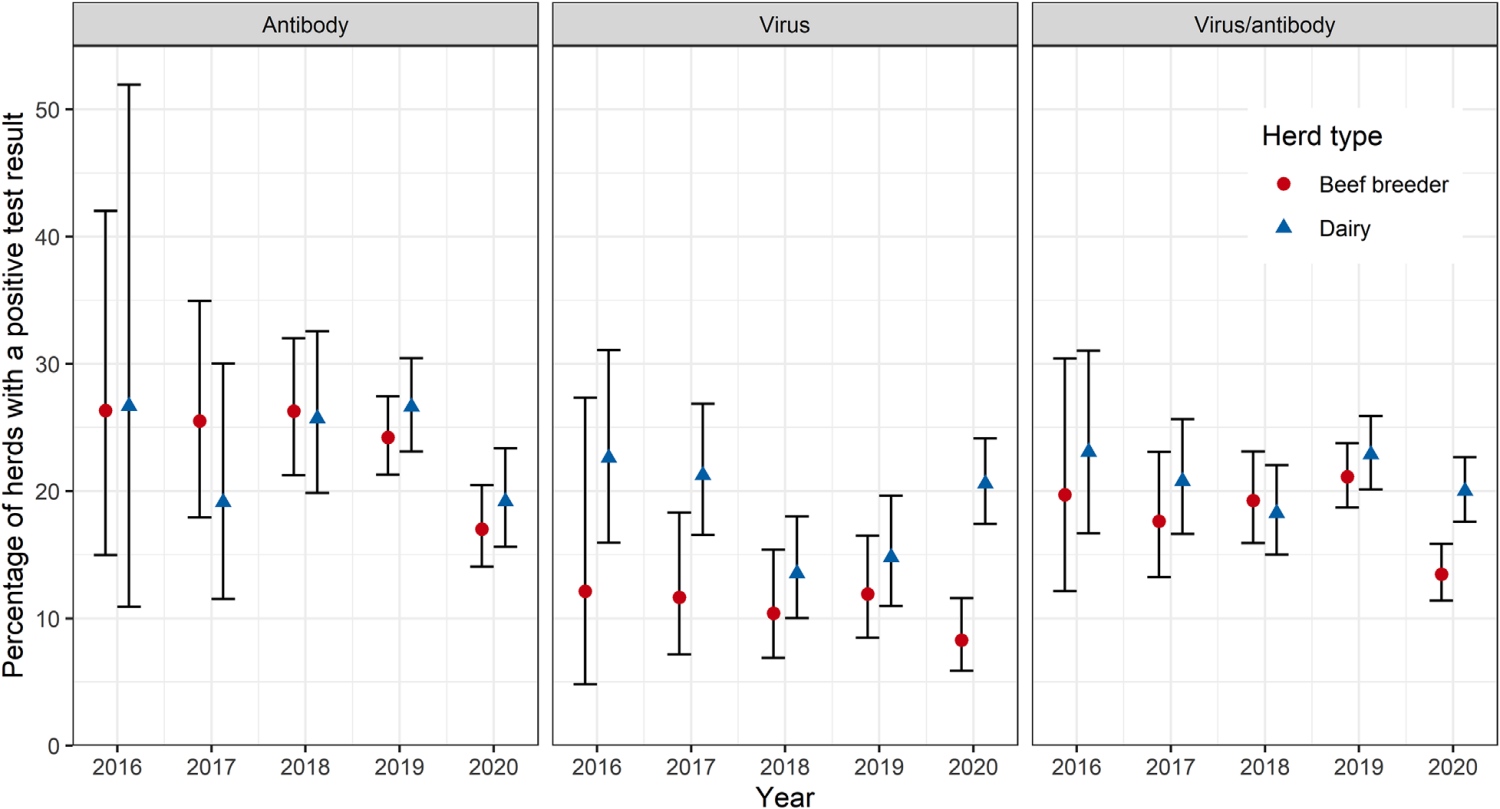
Percentage of antibody or virus testing beef breeder herds (red circles) or dairy herds (blue triangles) positive for bovine viral diarrhoea antibody or virus with binomial 95% confidence interval error bars

**FIGURE 4:**
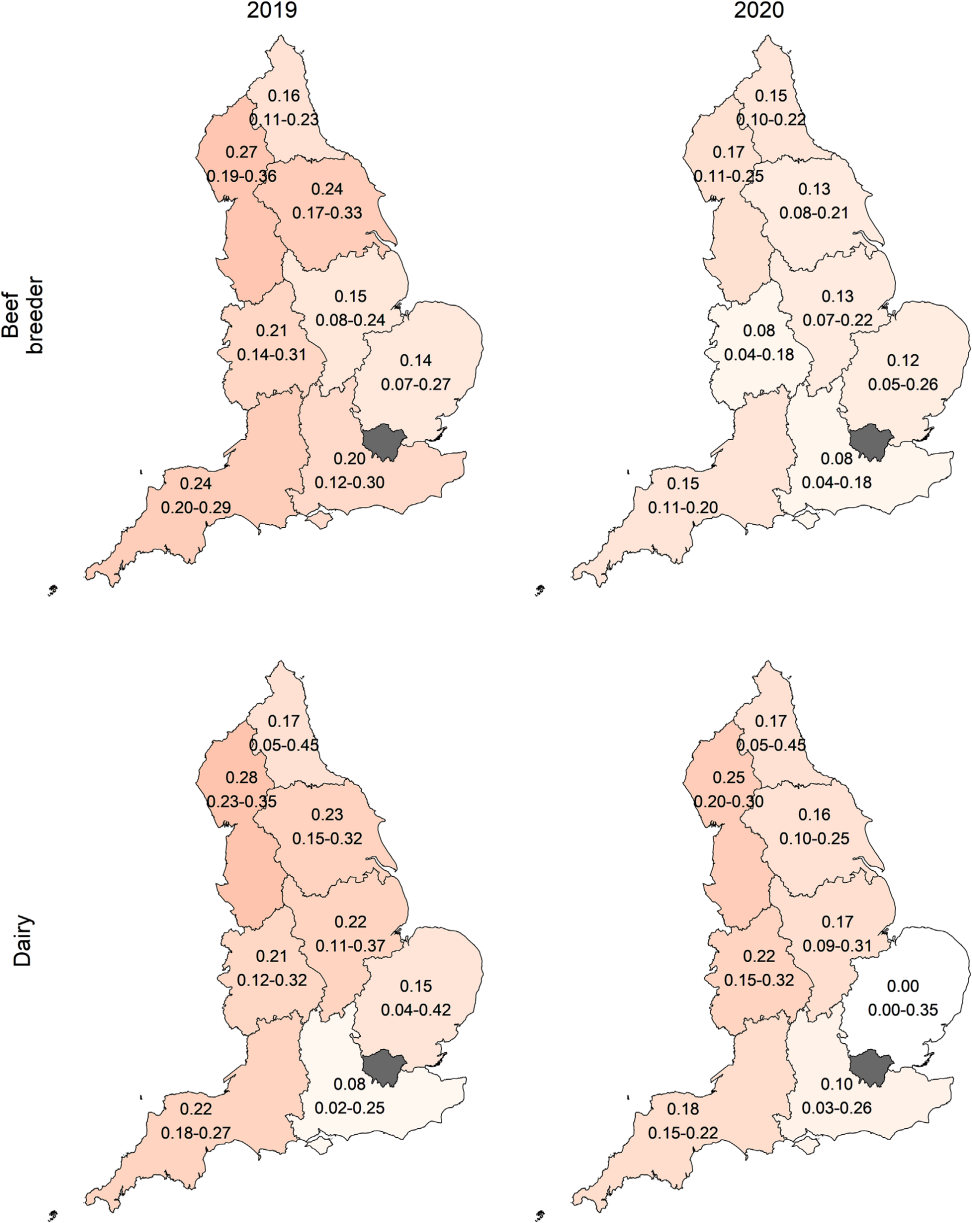
Proportion of beef breeding and dairy herds in each region of England testing positive for bovine viral diarrhoea (BVD) virus or antibody in 2019 and 2020, with binomial 95% confidence intervals below. Darker red shading corresponds to a higher proportion of herds testing positive for BVD virus or antibody

In 2020, 13.5% (120/891) of beef breeding herds had at least one positive individual virus or individual antibody test (8.3% [30/362] of virus testing herds and 17.0% [90/529] of antibody testing herds) compared to 20.0% (190/950) of dairy herds (20.6% [114/554] of virus testing herds and 19.2% [76/396] of antibody testing herds). There were fluctuations in the prevalence of positive herds between years with no clear trend (Figure 3). Among virus testing herds, there was a numerically higher (but not statistically different) prevalence of herds with positive test results in dairy herds than beef breeder herds for every year, but not for antibody testing herds (Figure 3). There were also differences in the prevalence of virus‐ or antibody‐positive herds by region between years (Figure 4). There were low numbers of farmers in some regions in earlier years of the BVDFree scheme, resulting in very wide confidence intervals; therefore, only the most recent years are shown.

### Prevalence of virus‐positive tests

**FIGURE 5:**
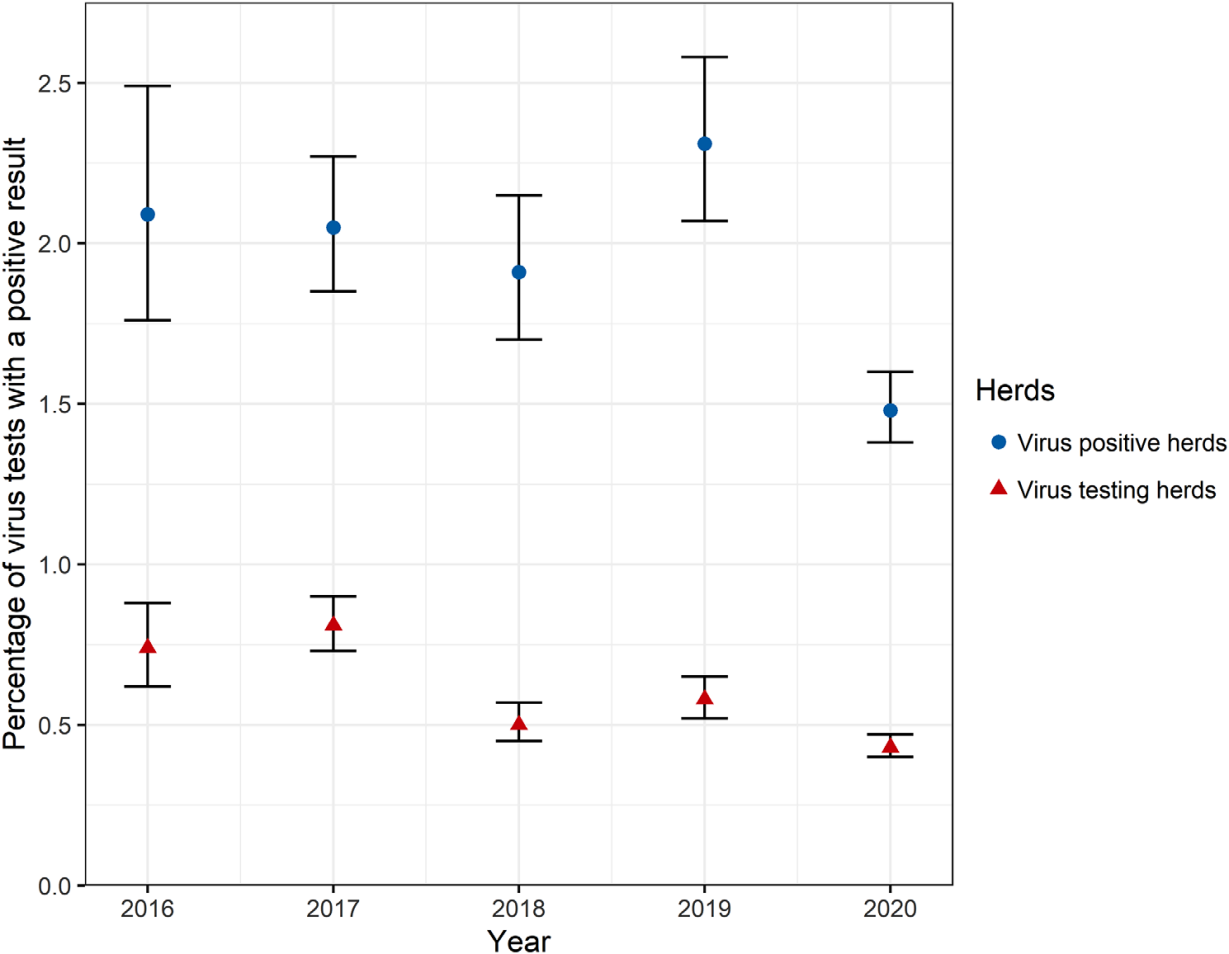
Percentage of individual bovine viral diarrhoea virus tests returning a positive result for all virus testing herds (red triangles) and virus‐positive herds (blue circles) each calendar year. Error bars represent the binomial 95% confidence interval

A total of 0.4% of tests submitted by virus testing herds in 2020 were positive, which was less than during the 2016–2019 period, where 0.5%–0.8% of tests submitted by virus testing herds were positive (Figure 5). Within virus‐positive herds, 1.5% of the tests submitted were positive in 2020, which was also less than that throughout 2016–2019 (Figure 5). However, regarding the herd‐level prevalence of positive tests, the animal‐level prevalence fluctuated between years with no clear trend.

### Prevalence of antibody‐positive tests

**FIGURE 6:**
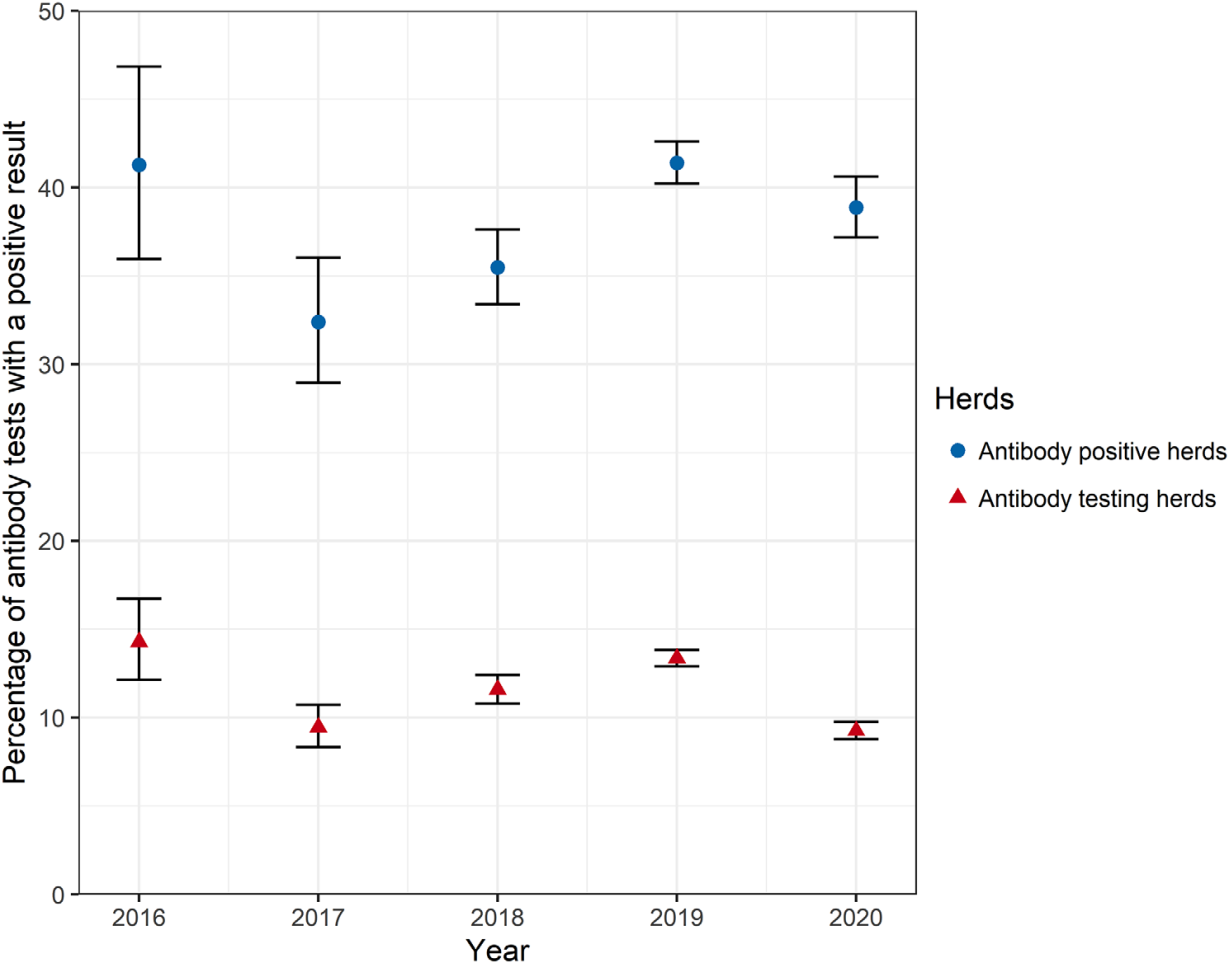
Percentage of individual bovine viral diarrhoea antibody tests returning a positive result for all antibody testing herds (red triangles) and antibody‐positive herds (blue circles) each calendar year. Error bars represent the binomial 95% confidence interval

A total of 9.3% of tests submitted by antibody testing herds in 2020 were positive, which was less than during the 2016–2019 period where between 9.5% and 14.3% of tests submitted by antibody testing herds were positive (Figure 6). Within antibody‐positive herds, 38.9% of the tests submitted were positive in 2020, compared to 32.4%–41.4% throughout 2016–2019 (Figure 6). There was also no clear temporal trend in the proportion of tests that were positive.

### Continued participation of herds in the scheme

In the generalised logistic mixed‐effects model, the likelihood of herds submitting tests to BVDFree the following year increased as the number of years they had submitted tests and the proportion of years they had received a positive test result increased. In addition, herds with higher numbers of breeding cows were also more likely to submit tests in the following year. Herds were also more likely to submit tests the following year if they submitted tests in 2017 and 2018 than 2016 and if they had tissue virus tested instead of blood antibody tested (Table 2). There was no association with herd type. Model fit was deemed adequate (Figure S4).

## DISCUSSION

In this paper, we present an analysis of the voluntary BVDFree England scheme's dataset of test results to assess current infection status, investigate the temporal trends in participation and identify associations between herd characteristics and the presence of BVD infection. Between 2016 and 2019, there was an increasing trend in the number of participating herds, with a small drop in the number of participating herds in 2020. Herd‐level prevalence was slightly higher in virus testing dairy herds than in beef breeder herds but not for antibody testing herds. We also identified a higher likelihood of participating again in the following year with increasing herd size in certain years and if a positive result had been previously reported for that herd.

A total of 13% of beef breeder herds were virus or antibody positive in 2020, compared to 20% of dairy herds. This is higher than the overall estimates of BVD prevalence in Northern Ireland and Ireland at the start of the compulsory phases of their schemes (11%)^14^, ^15^, ^17^ but similar to the prevalence in Ireland in 2012 at the beginning of their voluntary phase (12% for beef and 18% for dairy).^26^ Our estimates are lower than the estimated 26% of herds that are BVD positive in Wales, which is also still in the voluntary phase of its eradication programme but has recruited approximately 80% of herds.^24^, ^27^ Previous research found a lower prevalence of BVD in dairy herds in England than in Wales.^28^ Scotland's estimated prevalence of BVD‐positive herds at the start of their compulsory phase was 40%. However, as this included herds that did not comply with testing, the true prevalence is likely to have been lower.^13^ These prevalence estimates (except for Wales) include all farms; however, due to the voluntary nature of the BVDFree scheme, the estimates here are from a sample of herds, and there is likely to be a bias in the farmers who join the scheme. The regression model investigating factors associated with herds continuing to participate in the scheme in the following year suggested that herds that had positive test results were more likely to stay in the scheme. BVD‐positive herds in Northern Ireland were more likely to be BVD positive in the future,^15^ and this, in addition to the larger herd size of participating herds here, suggests that the prevalence of BVD‐positive herds in England could be lower than estimated in this study. However, we do not know if there is a bias for herds that are more or less likely to be BVD positive to join BVDFree. In Northern Ireland, there was a higher prevalence of BVD in the herds participating in the voluntary phase than when testing became mandatory for all herds,^15^ and it is possible that farmers who think they have herds with BVD may be more likely to join and test.

There was no clear trend in the prevalence of virus‐ or antibody‐positive herds, which fluctuated by year. The fluctuations could result from the large number of farmers recruited to the scheme in 2019 who had not engaged with the scheme previously and therefore may have been less likely to be controlling BVD (Figure 1) and changes in the farmers participating between years. Although not statistically significant, dairy herds usually had a slightly higher prevalence of BVD test‐positive herds in each year than beef breeder herds, which is common in the literature.^10^, ^17^, ^29^ However, the difference we identified is lower than that found in other studies,^10^ even for 2020, which had the largest difference in prevalence of positive tests between herd types. Dairy herds are expected to have a higher prevalence of BVD than beef herds due to the larger sizes of dairy herds increasing the transmission of infection (both larger herds and dairy herds are more likely to be BVD positive^15^, ^30^, ^31^) or due to the increased mixing of cows within dairy herds compared to beef herds.

There were fewer virus‐positive cattle in a herd in 2020 than in the previous 4 years, with 0.4% of cattle in virus testing herds positive for BVD. There was also a lower proportion of cattle positive for BVD virus in virus‐positive herds in 2020 (1.5%) than in the previous 4 years. This test positive rate is similar to other European countries, which typically have a BVD prevalence of less than 0.8% of animals,^10^ and lower than Northern Ireland, where 0.6% of calves were PIs in the first year of their compulsory phase.^29^ Similarly, 0.5% of calves in Ireland were virus positive at the start of their voluntary phase.^26^ We were unable to identify transiently infected cattle in the dataset because we did not have animal identification data to link an animal to a test. In Ireland, 83% of virus‐positive calves were confirmed to be PIs.^26^ Therefore, the actual PI rate may be lower than this estimate. There are typically low numbers of PI calves per positive herd, with only one PI detected in 66%–71% of BVD‐infected herds in Ireland and Northern Ireland.^26^, ^29^

Dairy herds were more likely to use virus testing than beef herds, and all herds were more likely to use antibody testing in 2018–2020 than in 2016. ‘Stamp It Out’ funding was available to veterinarians to carry out blood antibody testing from 2018 to 2021, which could have encouraged farmers to use antibody testing during those years. Dairy herds, being larger, are both the most likely to have sufficient youngstock numbers for blood antibody cohort testing and likely to find antibody testing more cost effective.^32^, ^33^ However, dairy herds may also be more limited in the choice of test, with calves not necessarily being retained on the farm past 9 months old making blood cohort antibody testing unfeasible, in contrast to beef breeding herds, which are more likely to have defined groups for antibody testing. Blood antibody testing must be carried out once calves are old enough (no younger than 6 months old) to avoid false positives from maternal antibodies, whereas tissue virus testing can be carried out on calves of any age.^34^ Virus testing dairy herds were more likely to be positive than virus testing beef breeder herds. It is possible that beef and dairy herds choose which test type to use for different reasons. Dairy herds have the option of bulk milk testing for BVD virus or antibodies, and it is possible that herds that already suspect they might have BVD through their bulk milk results use virus testing to directly identify the PI calves, whereas those who suspect that they are negative could find antibody testing more cost effective.

Herds that join the scheme do not always continue submitting tests in the following years. Continued submission of tests to the scheme was associated with years in which funding was available for testing, using virus testing, large herds and being virus or antibody positive in a previous year. An increase in participation coinciding with available funding is common to other BVD control schemes,^35^ and these results highlight that entirely voluntary BVD control will not engage a high enough number of farmers. The cost effectiveness of testing for BVD is lower in herds that do not have BVD^13^ and that are not experiencing production losses from BVDV infection, which may explain why farmers with negative test results are less likely to test the following year. Increased demand from buyers for BVD‐free accredited stock would help increase the benefits of testing to BVD‐free farmers. Antibody testing is more cost effective in larger herds since they have greater numbers of youngstock per management group, and this finding has also been identified in other voluntary BVD schemes,^36^ which may explain the over‐representation and increased continued participation of larger herds in the scheme. Virus testing herds were more likely to stay engaged with the BVDFree scheme than those using antibody testing. Often, a tissue tag test is incorporated into the individual animal identification tag that all calves require by law; therefore, tissue virus testing easily fits into the farmers’ routine, which could be one possible explanation. The need for research into ways of improving farmer engagement with BVD control has been identified previously.^37^ Most farmers join health schemes for their own benefits rather than contributing to national disease control.^35^ This issue is also compounded by the fact that not all BVD‐engaged herds submit tests to BVDFree because there are other accreditation schemes available.

A limitation of this study is that participating farmers are unlikely to be a representative sample of English cattle farmers, which is partially evidenced in the differences in herd sizes and types recruited to the scheme each year—herds in the scheme were 1.5–2 times larger than average.^24^ We do not know the rationale farmers have for joining the scheme. Research into the motivations of farmers to join a voluntary regional BVD scheme found no clear common reason, other than on veterinary advice.^36^ Dairy farmers are over‐represented in the dataset, which is similar to the situation during the voluntary phase in Ireland.^26^ It was not possible to ascertain from the dataset if farmers were following the BVDFree testing requirements correctly (i.e., tissue testing every calf or antibody testing the correct number of youngstock of the correct age per management group) and therefore what BVD status they would have been assigned by their veterinarian from their test results. The prevalence of BVD‐positive herds detected here may be underestimated because we do not know if the correct number of animals at the correct ages or for the required time period were tested. Consequently, some of the herds we assign as being BVD negative may not have been truly negative. Additionally, we categorised farmers by the tests they used in any one calendar year for ease of analysis and because we do not know when each farmer commenced and concluded each round of testing. Therefore, we may have classified some farmers by a testing regimen in a way that may not exactly reflect how the tests were used. Additionally, we do not know the identification numbers of tested animals, so we cannot identify which animals were suspected of being transiently infected and therefore retested. This information would be a valuable addition to this analysis. Finally, these findings reflect the specific funding, testing and support landscape for BVD control in England; however, some of the findings may be generalisable to other countries with voluntary schemes.

In conclusion, we have presented an estimate of the prevalence of BVD in England from a national dataset and find that although the prevalence of BVD‐positive tests in herds submitting test results to BVDFree was lower in 2020 than in previous years, there was not a clear decreasing trend in either the proportion of positive tests or positive herds over that time frame. Not all herds remained engaged in the scheme; herds with positive BVD tests, those using virus testing and larger herds were more likely to submit tests the following year, and continued engagement was also associated with available funding. More widespread farmer engagement appears crucial for the effective control of BVD in England, and research to stimulate this may be worthwhile.

## CONFLICTS OF INTEREST

Derek Armstrong and Lorna Gow are directly involved in the delivery of the BVDFree programme.

## ETHICS STATEMENT

Ethical approval was granted by the University of Warwick Biomedical and Scientific Research Ethics Committee (BSREC 100/19‐20) prior to commencement of the study.

## AUTHOR CONTRIBUTIONS

Martin Green, Michael Tildesley, Jasmeet Kaler, Eamonn Ferguson and Matt Keeling were responsible for funding acquisition and supervision. Derek Armstrong, Lorna Gow and Martin Green were involved in conceptualisation. Naomi Prosser, Martin Green and Edward Hill were involved in the formal analysis, investigation and methodology. Naomi Prosser curated the data and wrote the original draft with input from Edward Hill, and all authors contributed to the review and editing of the paper.

## Supporting information

Supporting Information

## Data Availability

Restrictions apply to the availability of these data, and interested parties should get in contact with BVDFree England. The data analysis code is available on request.
