# Supplementary material for: Descriptive analysis of national bovine viral diarrhoea test data in England (2016–2020)
Source: Vet Rec. 2022 Jul 25;191(5):e1854. doi: 10.1002/vetr.1854 (PMC9546236; doi:10.1002/vetr.1854)

Supplementary information for:

Descriptive analysis of national bovine viral diarrhoea test data in England

Naomi S. Prosser, Edward M. Hill, Derek Armstrong, Lorna Gow, Michael J. Tildesley, Matt J. Keeling, Jasmeet Kaler, Eamonn Ferguson, Martin J. Green

Supplementary Figure 1: Density curve of the number of individual tests carried out by antigen testing herds as a proportion of herd size for beef breeding (red solid line) and dairy herds (blue dashed line). The area under the curve equals 1.

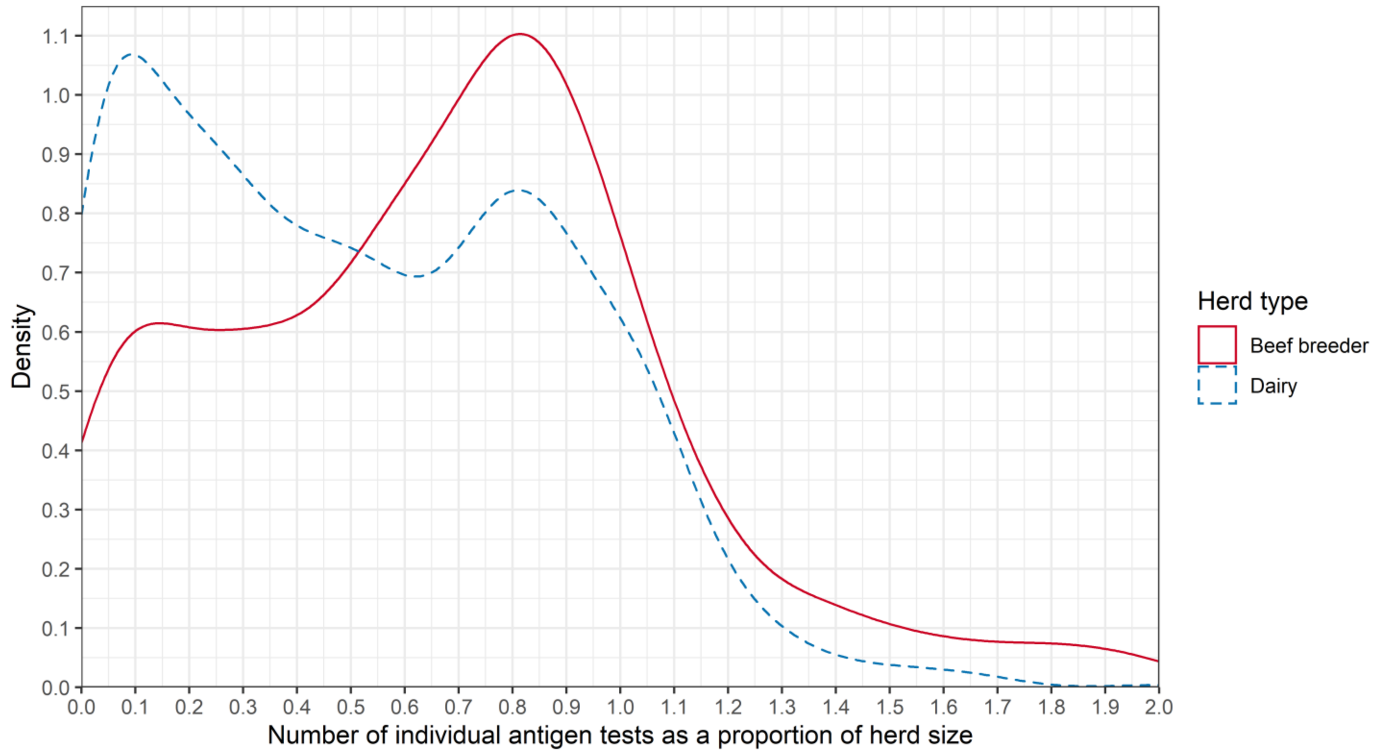

Supplementary Figure 2: Proportion of antigen testing herds that were positive for BVD at different cut-offs for the minimum proportion of the herd tested for beef breeder herds (red circles) and dairy herds (blue triangles).

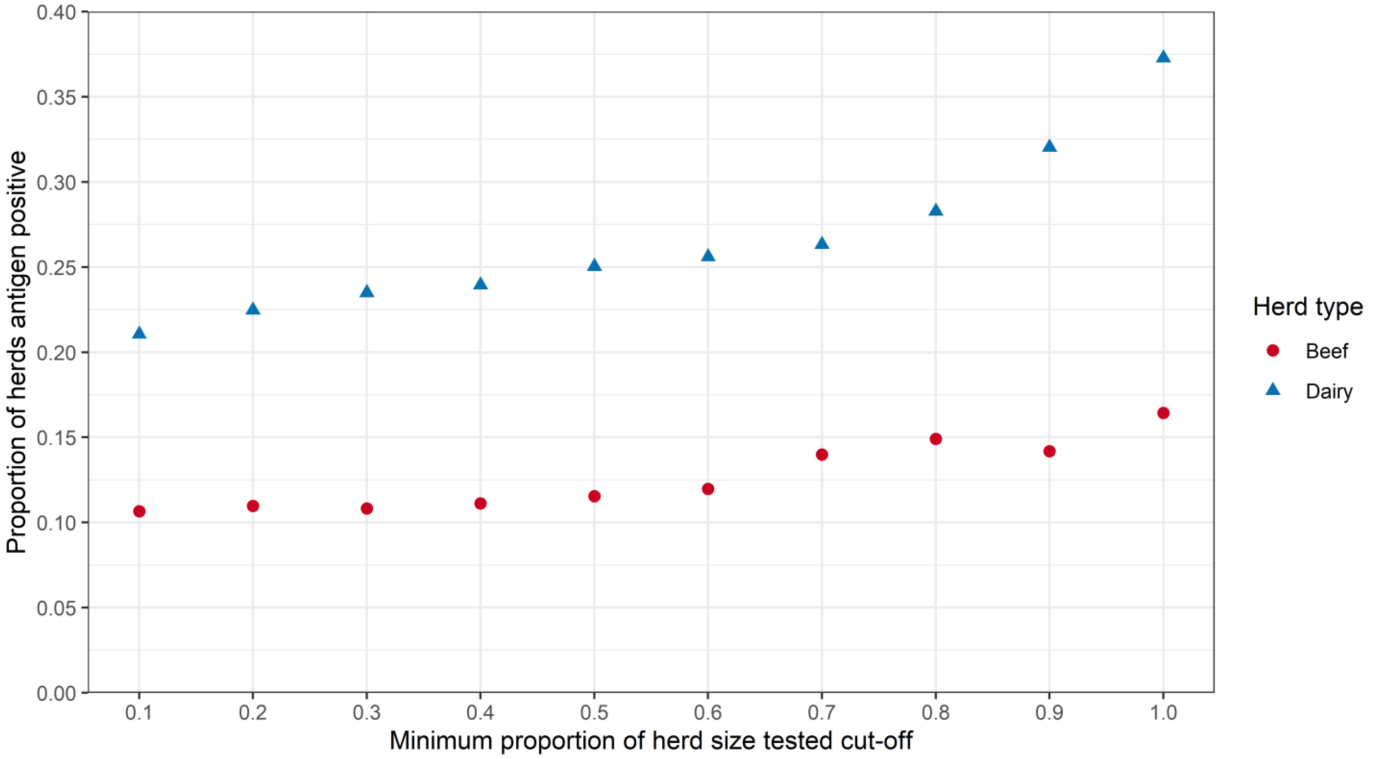

Supplementary Figure 3: Decile plot of the fit of a generalised logistic mixed-effects model explaining virus versus antibody test regime for 2,746 herds from 2016 to 2020, with 5,139 herd-years. Orange bars correspond to the observed data and blue bars correspond to the predictions from the generalised logistic mixed-effects model. Adequate fit is indicated by similar observed and predicted proportions for each decile.

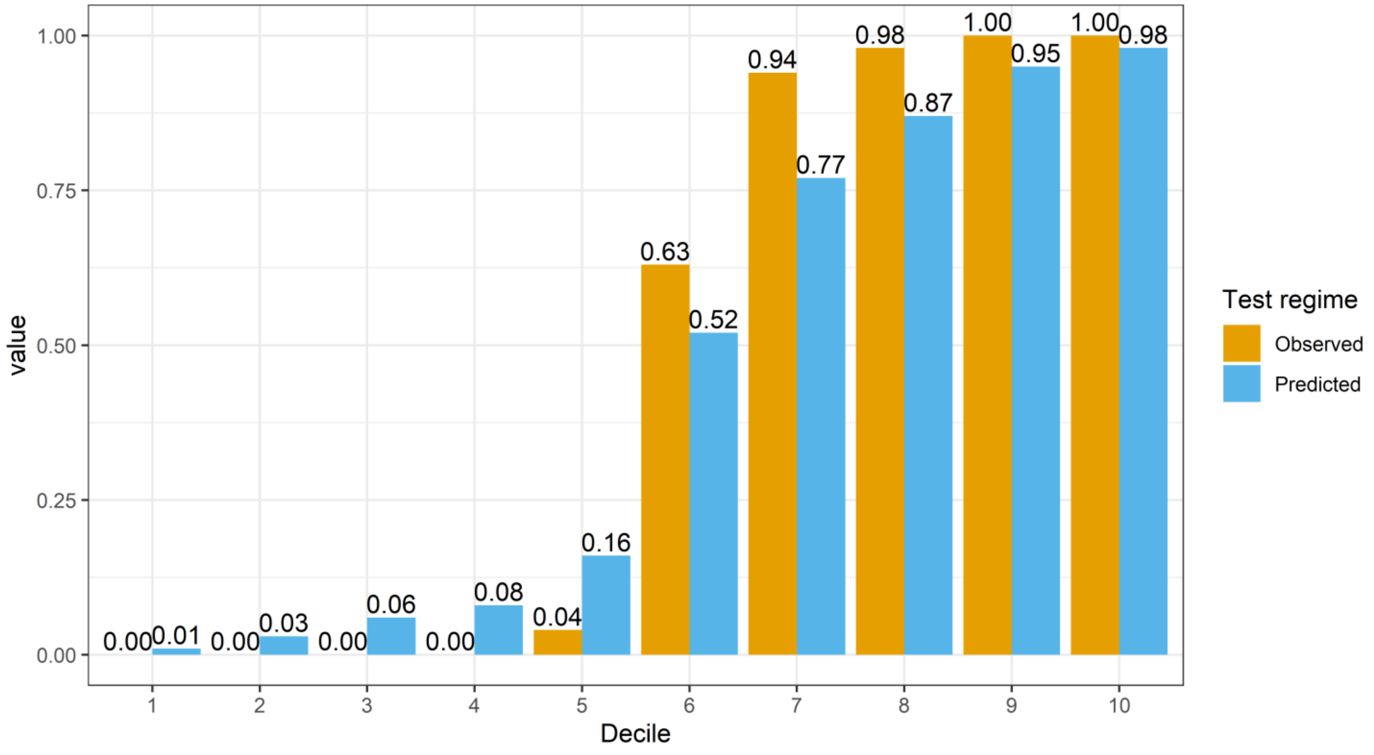

Supplementary Figure 4: Decile plot of the fit of a generalised logistic mixed-effects model explaining herds not submitting BVD tests the following year for 2,222 herds from 2016 to 2019, with 3,355 herd-years. Red bars correspond to the observed data and blue bars correspond to the predictions from the generalised logistic mixed-effects model. Adequate fit is indicated by similar observed and predicted proportions for each decile.

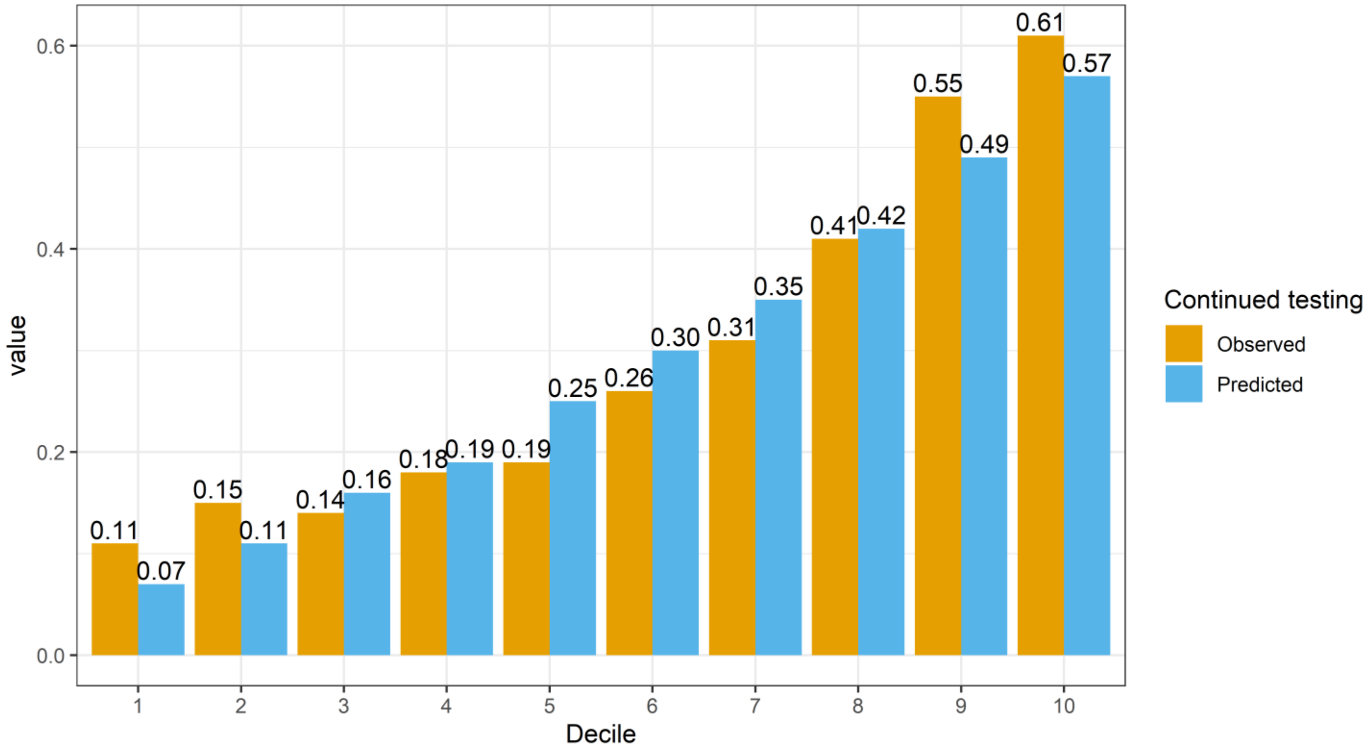

Supplement: Supplementary file 1 — Supporting Information [file VETR-191-no-s001.pdf]
